# Multiparametric MRI radiomics for preoperative prediction of tumor budding grade in rectal cancer

**DOI:** 10.3389/fonc.2026.1984365

**Published:** 2026-10-14

**Authors:** Peng Chen, Yanyan Zhou, Dan Wu, Chengmeng Zhang, Xiangyi He, Ru Pan, Dong Xia, Jinge Li, Jue Hou, Jian Shen, Hao Chen

**Affiliations:** 1 Department of Radiology, Huzhou Central Hospital, Fifth School of Clinical Medicine of Zhejiang Chinese Medical University, Huzhou, China; 2 Department of Radiology, Changxing Hospital of Traditional Chinese Medicine, Huzhou, China; 3 Department of Pathology, Huzhou Central Hospital, Fifth School of Clinical Medicine of Zhejiang Chinese Medical University, Huzhou, China; 4 School of Medicine and Nursing, Huzhou Normal University, Huzhou, China

**Keywords:** explainable artificial intelligence, machine learning, magnetic resonance imaging, radiomics, rectal cancer, tumor budding

## Abstract

**Background:**

Tumor budding (TB) is a histopathological marker of tumor aggressiveness and an established prognostic factor in colorectal cancer. In rectal cancer, however, TB is usually assessed on biopsy or resection material, and a reliable result may not be available when preoperative management is being considered.

**Purpose:**

To develop and internally validate a radiomics model for preoperative discrimination of low-grade versus intermediate/high-grade TB in patients with rectal cancer who proceed to upfront surgery without neoadjuvant treatment. The primary model was selected according to the highest repeated cross-validation AUC, with alternative feature sets and algorithms evaluated exploratorily.

**Methods:**

This retrospective study included 139 patients with pathologically confirmed rectal cancer who had received no neoadjuvant treatment. TB was assessed on postoperative resection specimens. T1WI, T2WI, arterial-phase contrast-enhanced T1WI, and DWI were analyzed. Radiomics features were extracted from the tumor core, a combined tumor-plus-2-mm expansion, and an isolated external 2-mm ring. Seven feature sets and four machine-learning algorithms were evaluated; the primary model was selected according to the highest repeated cross-validation AUC. Feature filtering, imputation, selection, and scaling were performed within the training portion of every split. Model performance was estimated using stratified five-fold cross-validation repeated five times and patient-level out-of-fold (OOF) probabilities.

**Results:**

The primary core-ROI Elastic Net model achieved a repeated cross-validation AUC of 0.825 (range 0.809–0.839 across repetitions), with PR-AUC 0.781, sensitivity 0.604, specificity 0.851, and Brier score 0.173. Its patient-level mean OOF AUC was 0.829 (2,000 paired bootstrap replicates; 95% CI, 0.755–0.896). At a probability threshold of 0.50, patient-level sensitivity was 0.607 and specificity was 0.867. In conditional paired bootstrap comparisons, the core model showed higher AUCs than the clinical, peritumoral-ring, clinical-plus-expanded, clinical-plus-core, and clinical-plus-core-plus-ring models (BH-adjusted P = 0.004–0.005). The difference versus the expanded ROI was smaller and did not reach statistical significance after adjustment (P = 0.052).

**Conclusion:**

Multiparametric MRI radiomics features from the tumor core showed promising internal discrimination of low-grade versus intermediate/high-grade TB among patients who underwent upfront surgery without neoadjuvant treatment. The model may support preoperative TB risk stratification in this population, but external validation is required before clinical use.

## Introduction

Rectal cancer is clinically heterogeneous even among patients with similar TNM stage. Differences in local invasion, lymph-node involvement, local vascular invasion, treatment response, recurrence risk, and survival are only partially captured by conventional anatomical staging. This heterogeneity has stimulated interest in biomarkers that reflect tumor biology and that can be measured before treatment. Such biomarkers are particularly relevant in rectal cancer because decisions regarding neoadjuvant therapy, total mesorectal excision, organ preservation, and surveillance are frequently made before definitive pathology is available (1, 2).

Tumor budding is defined by isolated single tumor cells or small clusters of up to four or five cells at the invasive front of the tumor. It is commonly interpreted as a morphological manifestation of tumor-cell dissociation, epithelial–mesenchymal transition, and local invasive behavior. Studies performed under the International Tumor Budding Consensus Conference framework have demonstrated that intermediate/high-grade TB is associated with lymph-node metastasis and adverse oncological outcomes. Recent pathological work has also emphasized the relationship between TB, poorly differentiated clusters, and other patterns of aggressive colorectal carcinoma biology (1–4).

The clinical challenge is that TB is most robustly assessed on resection specimens. Biopsy-based assessment is vulnerable to sampling error because the invasive front may not be represented in a small tissue fragment. Furthermore, TB may be spatially heterogeneous within the same tumor. A noninvasive imaging surrogate could therefore provide preoperative information that complements pathological assessment rather than replacing it. In clinical practice, even an imperfect imaging estimate may help identify patients for closer multidisciplinary review or additional risk stratification (5).

MRI is the standard imaging modality for local staging of rectal cancer. High-resolution T2-weighted imaging depicts the relationship between the primary lesion, muscularis propria, mesorectal fascia, and adjacent structures. Diffusion-weighted imaging provides information related to water mobility and cellular organization, while contrast-enhanced T1-weighted imaging may reflect vascularity, permeability, and stromal composition. T1-weighted images can contribute anatomical and signal-intensity information. These sequences therefore provide complementary views of the tumor and its microenvironment (6, 7).

Radiomics converts medical images into quantitative descriptors of intensity distribution, shape, and texture. MRI radiomics has been explored for lymph-node metastasis, pathological complete response, treatment response, recurrence, microsatellite instability, and other clinically relevant endpoints in rectal cancer. Importantly, several recent studies have specifically examined TB prediction. MRI-based radiomics has been reported as feasible for presurgical evaluation of TB in locally advanced rectal cancer (8). T2-weighted radiomics combined with machine-learning methods has also been used to predict TB grade preoperatively (9). Other studies have investigated b-value threshold maps (10), multiparametric MRI radiomics (11), diffusion-related biomarkers such as IVIM-DWI (12, 13), multimodal transfer learning (14), Vision Transformer models (15), attention-based ensemble learning (16), and APT-weighted MRI (17). A recent systematic review and meta-analysis further summarized the growing evidence base for MRI-based TB prediction (18).

Most radiomics studies focus on the tumor itself. However, TB is located at the invasive front, where tumor cells interact with stroma, inflammatory cells, extracellular matrix, and mesorectal tissue. A peritumoral region may therefore capture imaging manifestations of desmoplastic reaction, microscopic extension, inflammatory infiltration, and boundary irregularity. The optimal definition of this region remains uncertain. A combined expansion includes the original tumor and the surrounding tissue, whereas an isolated external ring captures only the peritumoral component. Comparing these definitions may provide insight into whether the signal is predominantly intratumoral, peritumoral, or distributed across the tumor boundary (6, 7).

The present study sought to determine whether multiparametric MRI radiomics could distinguish rectal cancer patients with low-grade from those with intermediate or high-grade TB by comparing intratumoral, expanded, and isolated peritumoral representations across four MRI sequences. We hypothesized that peritumoral information might add discriminatory value, but did not assume that any spatial definition would be superior. We evaluated seven clinically motivated feature sets, four machine-learning algorithms, and complementary analyses of calibration, clinical utility, and model explanation. The repeated cross-validation AUC was used to select the primary model.

## Methods

### Study design and patient selection

This retrospective single-center study was conducted at Huzhou Central Hospital from February 2023 to June 2026. The study was approved by the Ethics Committee of Huzhou Central Hospital (approval number: 2026-177-01). The requirement for written informed consent was waived because of the retrospective design. All 139 patients underwent surgery without neoadjuvant treatment, and the interval between MRI and surgery was within 1 week.

Eligibility criteria were: (1) pathologically confirmed rectal adenocarcinoma; (2) preoperative MRI containing T1WI, T2WI, contrast-enhanced T1WI, and DWI; (3) available tumor-budding classification; and (4) available baseline clinical information. Exclusion criteria were: (1) incomplete or unavailable MRI data, or severe image artifacts; and (2) receipt of neoadjuvant treatment before surgery. Patients receiving neoadjuvant treatment were excluded because treatment may alter the tumor budding observed in the resection specimen, making this postoperative reference standard non-equivalent to the untreated tumor phenotype represented by pretreatment MRI. The study work flow is shown in **Figure 1**.

**Figure 1:**
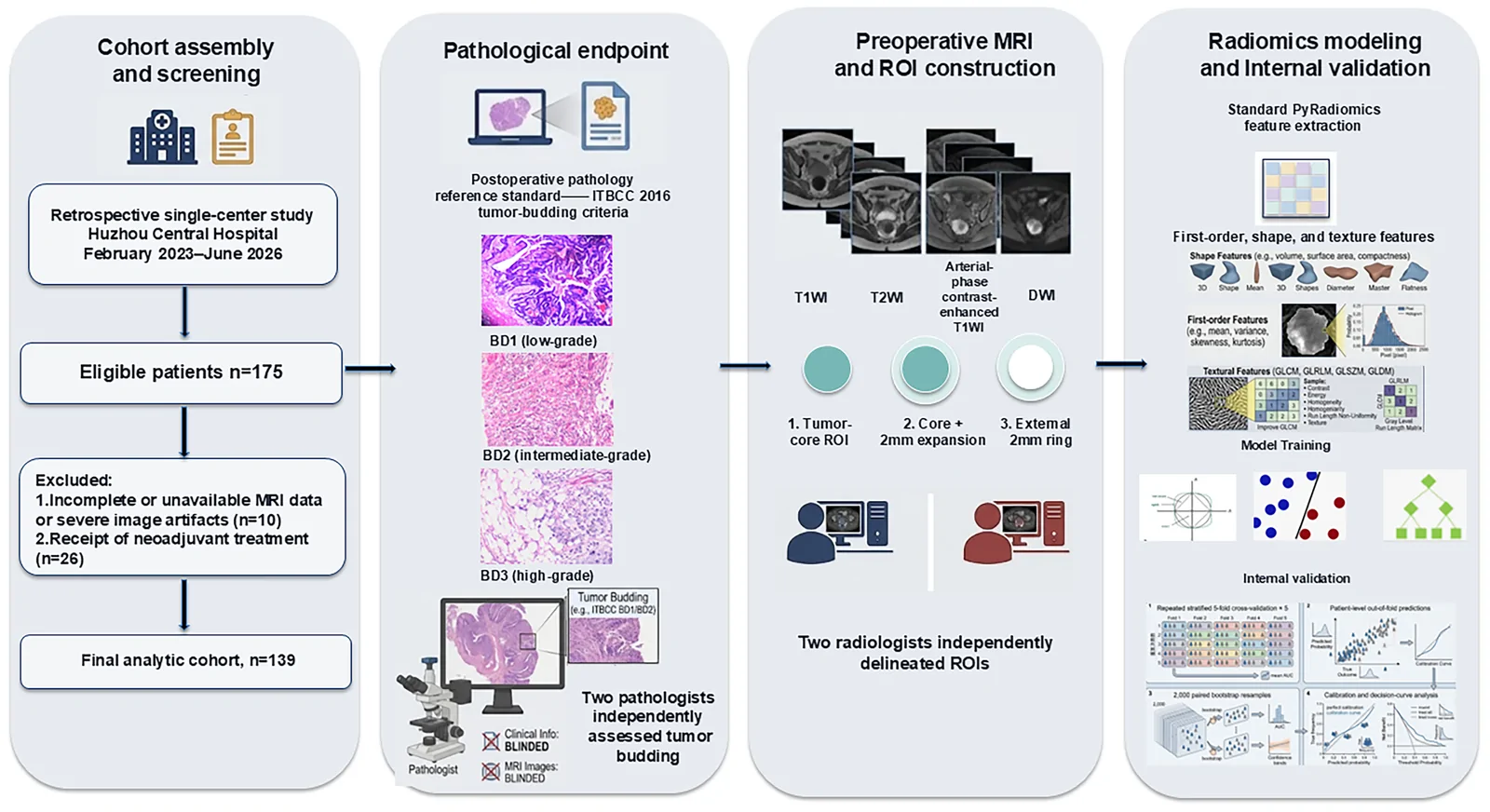
Study workflow.

### Definition of pathological TB grading

The binary endpoint was derived from postoperative pathology using the 2016 International Tumor Budding Consensus Conference (ITBCC) criteria (5). Hematoxylin-and-eosin-stained sections were independently reviewed by two pathologists, one with intermediate professional rank and more than 5 years of experience and one senior pathologist with more than 20 years of experience. Both pathologists were blinded to MRI and clinical data. A hotspot at the tumor invasive front was selected, and buds were counted under a 20× objective (field area, 0.785 mm²). A tumor bud was defined as a single tumor cell or a cluster of no more than four tumor cells within the invasive-front stroma. BD1 was defined as 0–4 buds, BD2 as 5–9 buds, and BD3 as ≥10 buds. BD1 was classified as low-grade TB, whereas BD2 and BD3 were combined as intermediate/high-grade TB. Discrepancies were resolved by joint re-review and consensus. Interobserver agreement for the independent pathology assessment was Cohen’s κ=0.92 (>0.90).

### MRI acquisition

All patients underwent preoperative pelvic MRI on a GE Signa HDxt 3.0-T system. The protocol included T1WI, T2WI, DWI, and multiphase contrast-enhanced T1WI. Representative parameters were as follows: T1WI, TR 710 ms and slice thickness/gap 5.0/1.0 mm; T2WI, TR/TE 2859/120 ms and slice thickness/gap 3.0/0.3 mm; DWI, TR/TE 6000/70–80 ms with b values of 0 and 800 s/mm² and slice thickness/gap 5.0/1.0 mm; and contrast-enhanced T1WI, TR/TE 4.1 ms/1.2 and 2.5 ms, flip angle 13°, and slice thickness/gap 2.0/1.08 mm. Gadopentetate dimeglumine was administered at 0.1 mmol/kg at 2.0–2.5 mL/s followed by a 20–30 mL saline flush. Four phases were acquired, including precontrast, arterial, portal venous, and delayed phases. The arterial phase was used for contrast-enhanced T1WI feature extraction in the primary analysis.

### ROI definitions and image quality control

Four sequence-specific tumor masks were available for T1WI, T2WI, contrast-enhanced T1WI, and DWI. Two radiologists independently delineated the ROIs slice by slice in 3D Slicer while blinded to the pathology results. The tumor-core ROI was manually delineated; the expanded ROI was generated by a 2-mm outward expansion in physical space and included the core; and the isolated ring ROI consisted only of the external 2-mm component after subtraction of the core. Inter-reader ROI reproducibility was ICC = 0.86 (>0.80). When the two radiologists disagreed, the final ROI was adjudicated by a third senior radiologist with 20 years of work experience, and the adjudicated mask was used for feature extraction. This ICC quantified inter-reader reproducibility of the manually delineated ROI masks between the two radiologists.

### Radiomics feature extraction and clinical variables

Radiomics features were extracted from the original image type using first-order, shape, gray-level co-occurrence matrix (GLCM), gray-level run length matrix (GLRLM), gray-level size zone matrix (GLSZM), gray-level dependence matrix (GLDM), and neighboring gray tone difference matrix (NGTDM) feature families. Across the 12 sequence–ROI combinations, 1,284 radiomics features were extracted, supplemented by baseline clinical variables. Before feature extraction, the multiphase MRI volumes underwent voxel-based rigid registration for spatial alignment using a prespecified reference volume, followed by preprocessing resampling to an isotropic voxel spacing of 1 × 1 × 1 mm³. B-spline interpolation was used for images and nearest-neighbor interpolation was used for ROI masks. Image intensities were normalized using nonzero voxels: m=median(x), s=1.4826×MAD(x), and x’=(x−m)/s; when the MAD-based scale was unavailable, the standard deviation was used as a fallback. Texture quantization used a fixed gray-level bin width of 25. During the final PyRadiomics extraction, no additional voxel resampling was applied and the preprocessed spacing was retained. No wavelet or Laplacian-of-Gaussian filtered image classes were included in the primary analysis. Candidate clinical variables included age, sex, BMI, tumor length, tumor distance from the anal verge, CEA, CA19-9, clinical T stage, clinical N stage, and tumor location.

### Feature sets

Seven feature sets were retained for the comparative analysis. The clinical set contained clinical variables only. The core set contained radiomics features from the intratumoral core. The expanded set contained features from the combined core-plus-2-mm expansion, whereas the ring set contained features from the isolated external 2-mm ring. The clinical-core set combined clinical variables with core radiomics features. The clinical-expanded fusion set combined clinical variables with expanded-region radiomics. The clinical-core-ring fusion set combined clinical variables with core and ring radiomics. The primary model was selected according to the highest repeated cross-validation AUC; all other model-feature-set combinations were treated as exploratory.

### Preprocessing, feature selection, and model fitting

All preprocessing steps were performed within the training portion of each cross-validation split. Features with no finite values were removed. Missing values were imputed using the training-set median. Features were standardized using the training-set mean and standard deviation. Candidate variables were ranked by the absolute correlation between the training feature and the binary outcome. Up to 10 clinical variables were retained for the clinical model. For radiomics-only models, up to 20 radiomics features were retained. For fusion models, clinical variables were retained and up to 20 radiomics variables were added. Highly redundant selected variables were filtered at an absolute pairwise correlation threshold greater than 0.95.

Elastic Net logistic regression used alpha=0.5 and selected lambda by the one-standard-error rule within the training data. Random forest used 300 trees, a minimum node size of five, and the square-root rule for mtry. XGBoost used shallow trees with maximum depth 2, learning rate 0.03, 150 boosting rounds, subsampling and column sampling of 0.8, and L1/L2 regularization. The SVM used a radial-basis-function kernel with cost 1 and gamma equal to the inverse of the number of input variables. These model specifications were fixed before the final rerun.

### Statistical analysis

All statistical analyses were performed using R software (version 4.5.1), and radiomics features were extracted using PyRadiomics (version 3.1.0). Baseline clinical characteristics were compared between the low-grade and intermediate/high-grade tumor-budding groups. Continuous variables were summarized as median (interquartile range) and compared using the Mann–Whitney U test. Categorical variables were summarized as number (percentage) and compared using the chi-square test or Fisher exact test, as appropriate.

The primary metric was the mean AUC across five repetitions of stratified five-fold cross-validation; empirical 2.5th–97.5th percentile ranges were reported as dispersion ranges rather than confidence intervals. Patient-level mean out-of-fold probabilities were used for calibration and decision-curve analysis. After selecting the model with the highest repeated cross-validation AUC, feature contributions were summarized using the full-cohort refit of the selected model. Paired patient-level bootstrap resampling with 2,000 iterations was used to estimate 95% confidence intervals and pairwise AUC differences among the seven retained Elastic Net feature-set models. Two-sided bootstrap P values were calculated with an add-one correction and adjusted using the Benjamini–Hochberg method across the 21 pairwise comparisons. Because model and feature-set selection used the same repeated cross-validation results and no nested cross-validation was performed, the performance of the selected primary model was treated as an exploratory internal estimate. A two-sided P value <0.05 was considered statistically significant.

## Results

### Cohort characteristics

Among 175 eligible patients, 36 were excluded: 10 because MRI data were incomplete or unavailable or severe image artifacts were present, and 26 because the patients had received neoadjuvant treatment before surgery. The final analytic cohort comprised 139 patients, including 83 low-grade TB cases and 56 intermediate/high-grade TB cases.

Baseline characteristics stratified by TB category are presented in **Table 1**. Tumor length, clinical T stage, and CEA differed between the low-grade and intermediate/high-grade TB groups, whereas the remaining reported variables did not show statistically significant differences.

**Table 1:** Baseline clinical characteristics of the study cohort according to tumor budding grade.

Variable	Low-grade TB (0), n=83	Intermediate/high-grade TB (1), n=56	P value
Age, years	68.0 (61.0–76.0)	71.0 (63.0–78.0)	0.150
Male sex	48 (57.8)	33 (58.9)	0.898
BMI, kg/m²	22.6 (20.0–24.6)	21.5 (20.0–25.0)	0.691
Tumor location			0.326
Low rectum	26 (31.3)	18 (32.1)	
Middle rectum	49 (59.0)	28 (50.0)	
High rectum	8 (9.6)	10 (17.9)	
Distance from anal verge, mm	63.5 (45.5–84.0)	64.5 (43.0–86.2)	0.803
Tumor length, mm	43.0 (33.0–54.0)	50.5 (41.8–65.0)	0.010
Clinical T stage			0.011
T1	2 (2.4)	1 (1.8)	
T2	29 (34.9)	9 (16.1)	
T3	48 (57.8)	36 (64.3)	
T4	4 (4.8)	10 (17.9)	
Clinical N stage			0.099
N0	26 (31.3)	9 (16.1)	
N1	41 (49.4)	31 (55.4)	
N2	16 (19.3)	15 (26.8)	
N3	0 (0.0)	1 (1.8)	
CEA, ng/mL	3.3 (2.3–8.2)	5.7 (3.1–12.4)	0.029
CA19-9, U/mL	9.2 (4.3–15.6)	8.7 (3.7–23.9)	0.987

Values are n (%) for categorical variables and median (interquartile range) for continuous variables. P values were calculated using the Mann–Whitney U test for continuous variables and Fisher exact test or χ² test for categorical variables, as appropriate. TB, tumor budding; BMI, body mass index; CEA, carcinoembryonic antigen; CA19-9, carbohydrate antigen 19-9.

### Comparison across feature sets and algorithms

As shown in **Figure 2A**, the core ROI feature set had the highest repeated cross-validation AUC across the evaluated algorithms. The core Elastic Net model achieved an AUC of 0.825, followed by core SVM (0.798). In the patient-level paired bootstrap comparison of the seven retained Elastic Net feature sets, the core model showed higher AUCs than the clinical, peritumoral-ring, clinical-plus-expanded, clinical-plus-core, and clinical-plus-core-plus-ring models (BH-adjusted P = 0.004–0.005; **Supplementary Table S1**). Its difference from the expanded ROI was smaller and did not remain statistically significant after adjustment (BH-adjusted P = 0.052).

**Figure 2:**
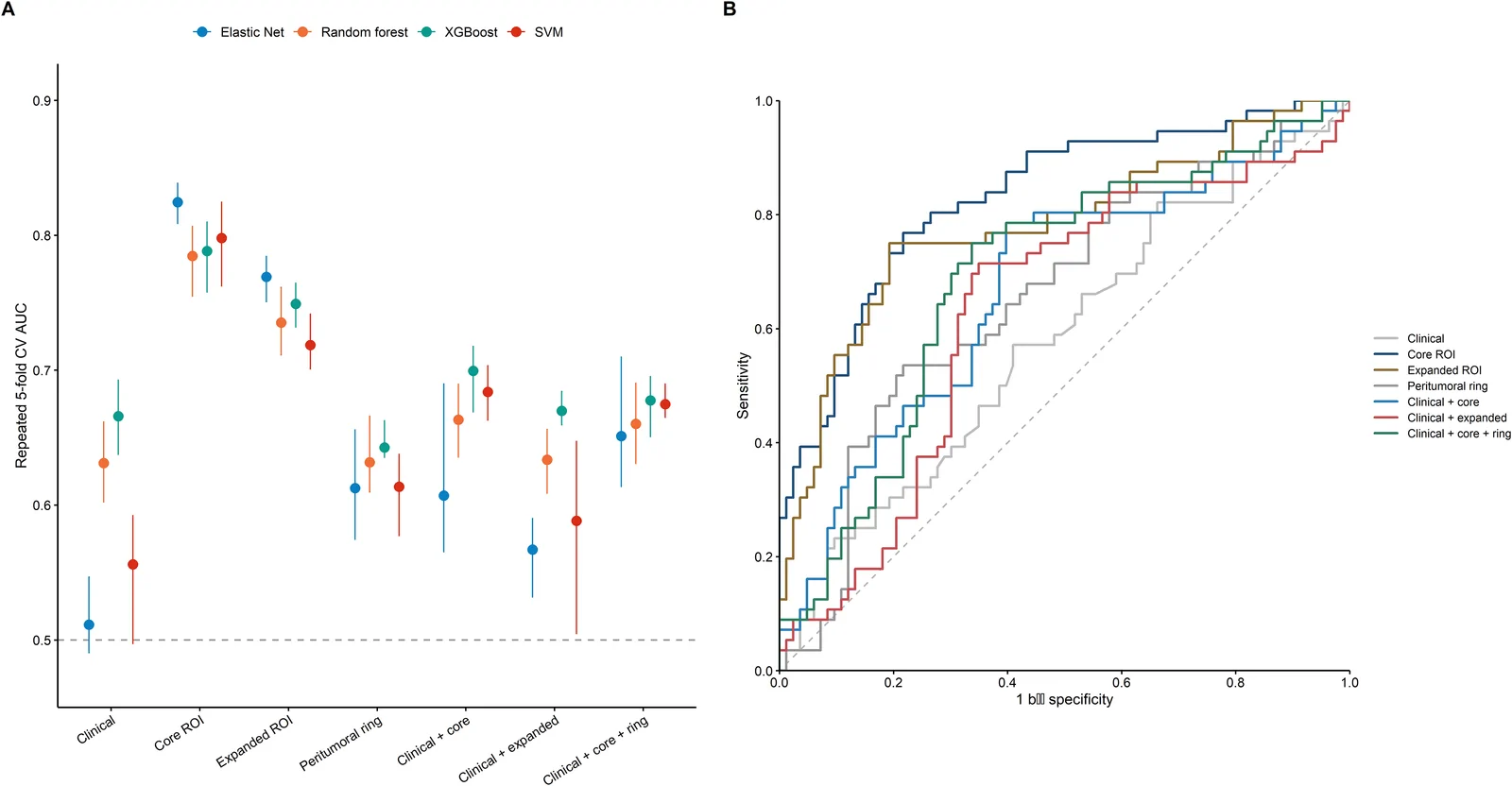
Model discrimination across feature sets and algorithms. **(A)** Repeated five-fold cross-validation AUCs for seven feature sets across Elastic Net, random forest, XGBoost, and SVM. **(B)** Patient-level mean OOF ROC curves for the seven Elastic Net feature-set models.

Clinical variables alone produced substantially weaker discrimination than core radiomics for Elastic Net and XGBoost. The isolated ring representation was informative but less discriminative than the tumor core. The expanded and fusion representations did not show a consistent incremental benefit. This pattern suggests that the strongest signal in the present data was associated with intratumoral heterogeneity and morphology rather than the narrow external ring. The complete model comparison is presented in **Table 2**.

**Table 2:** Repeated cross-validation performance of models using seven retained feature sets.

Feature set	Elastic net	Random forest	XGBoost	SVM
Clinical	0.511 (0.491–0.547)	0.631 (0.602–0.662)	0.666 (0.638–0.693)	0.556 (0.497–0.592)
Core ROI	0.825 (0.809–0.839)	0.785 (0.755–0.807)	0.788 (0.758–0.810)	0.798 (0.762–0.825)
Expanded ROI	0.769 (0.751–0.784)	0.735 (0.711–0.761)	0.749 (0.732–0.765)	0.719 (0.701–0.742)
Peritumoral ring	0.613 (0.575–0.656)	0.632 (0.610–0.666)	0.643 (0.635–0.663)	0.614 (0.577–0.638)
Clinical + expanded	0.568 (0.535–0.599)	0.635 (0.612–0.672)	0.673 (0.664–0.684)	0.615 (0.568–0.655)
Clinical + core + ring	0.626 (0.554–0.680)	0.655 (0.641–0.681)	0.672 (0.654–0.687)	0.684 (0.656–0.719)
Clinical + core	0.607 (0.565–0.690)	0.663 (0.636–0.690)	0.700 (0.669–0.718)	0.684 (0.663–0.703)

Values are mean AUC with the empirical 2.5th–97.5th percentile range across five repeated five-fold cross-validation estimates. PR-AUC, area under the precision–recall curve; CV, cross-validation; ROI, region of interest; SVM, support vector machine.

### Primary model performance

The selected primary core Elastic Net model achieved the highest mean repeated cross-validation AUC of 0.825, with an empirical 2.5th–97.5th percentile range of 0.809–0.839. The corresponding PR-AUC was 0.781, sensitivity was 0.604, specificity was 0.851, and the Brier score was 0.173. Based on patient-level mean out-of-fold probabilities, the model achieved an AUC of 0.829 (95% CI, 0.755–0.896) after 2,000 paired bootstrap replicates. These results indicate promising internal discrimination, although the confidence interval remained broad and does not represent external validation. **Figure 2B** presents patient-level OOF ROC curves for the seven retained Elastic Net feature-set models. The core curve showed favorable visual separation from the clinical-only, clinical-plus-core, ring-only, expanded, and other retained fusion curves, although all curves came from the same internally validated cohort.

### Patient-level classification and calibration

At a probability threshold of 0.50, the primary model classified 45 patients as intermediate/high-grade TB and 94 as low-grade TB. The confusion matrix comprised 34 true positives, 72 true negatives, 11 false positives, and 22 false negatives. Accuracy was 0.763, sensitivity 0.607, specificity 0.867, positive predictive value 0.756, negative predictive value 0.766, and F1 score 0.673. These values are summarized in **Table 3** and **Figure 3A**. Because 0.50 was used as a conventional descriptive threshold rather than a clinically optimized cutoff, sensitivity and specificity were also examined at thresholds of 0.30, 0.40, and 0.50: 0.911/0.506, 0.768/0.771, and 0.607/0.867, respectively. **Figure 3B** showed a generally monotonic calibration pattern, although some deviation from ideal calibration remained.

**Table 3:** Patient-level classification performance of the primary core Elastic Net model.

Metric	Estimate
N	139
TP/TN/FP/FN	34/72/11/22
Accuracy	0.763
Sensitivity	0.607
Specificity	0.867
PPV	0.756
NPV	0.766
F1 score	0.673

Metrics were calculated from mean patient-level OOF probabilities at a threshold of 0.5. TP, true positive; TN, true negative; FP, false positive; FN, false negative; PPV, positive predictive value; NPV, negative predictive value.

**Figure 3:**
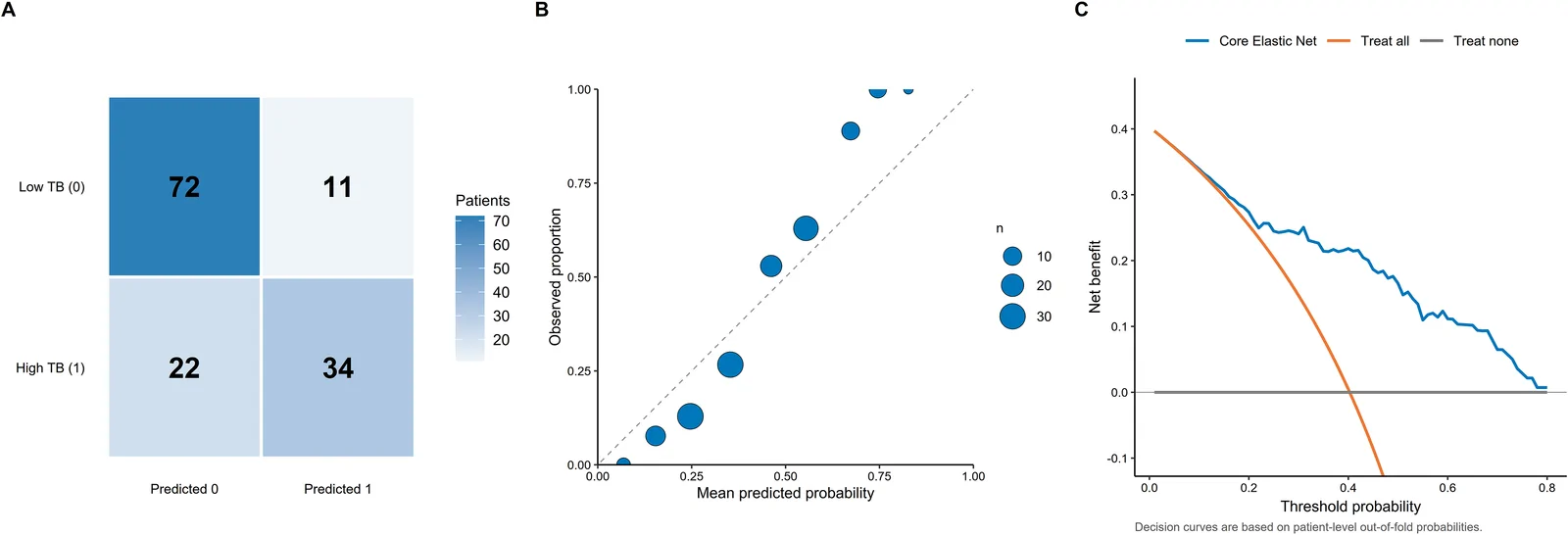
Internal validation and clinical utility of the primary model. **(A)** Patient-level confusion matrix for the core Elastic Net model at a probability threshold of 0.50. **(B)** Calibration curve based on patient-level mean OOF probabilities. **(C)** Decision-curve analysis across thresholds 0.01–0.99, comparing the core Elastic Net model with treat-all and treat-none strategies. Threshold 0.50 was used for descriptive classification only and was not selected as a clinically optimized cutoff.

### Decision-curve analysis

**Figure 3C** shows the DCA. The analysis evaluated thresholds from 0.01 to 0.99. The core model had net benefit above treat-all from 0.05 to 0.82 and positive net benefit relative to treat-none from 0.01 to 0.82. Representative net benefits for the core model at thresholds 0.10, 0.20, 0.30, 0.40, and 0.50 were 0.3389, 0.2734, 0.2405, 0.2182, and 0.1655, respectively. In practice, a positive result would prompt closer multidisciplinary review rather than, on its own, changing the neoadjuvant treatment or surgical plan.

### Feature explanation and radiomics phenotype

The four leading explanatory features in the full-cohort refit of the selected core Elastic Net model were T2WI core sphericity, T1WI core sphericity, contrast-enhanced T1WI core GLCM maximal correlation coefficient (MCC), and contrast-enhanced T1WI core NGTDM strength (**Figure 4** and **Table 4**). Across the 25 training folds, these variables passed the candidate-screening step in 25, 25, 23, and 17 folds, respectively (**Supplementary Table S4**). These counts describe internal training-fold screening only; they do not indicate that each feature had a nonzero Elastic Net coefficient in that many folds. Mean absolute contribution values are shown in **Figure 4A**, while **Figure 4B** displays the direction and magnitude of coefficient-derived contributions from the full-cohort refit. Positive contributions increase the fitted log-odds of intermediate/high-grade TB, whereas negative contributions reduce them. **Figure 4C** shows standardized feature values across the two TB groups. The four displayed variables are explanatory features of the fitted model, not confirmed stable biomarkers.

**Figure 4:**
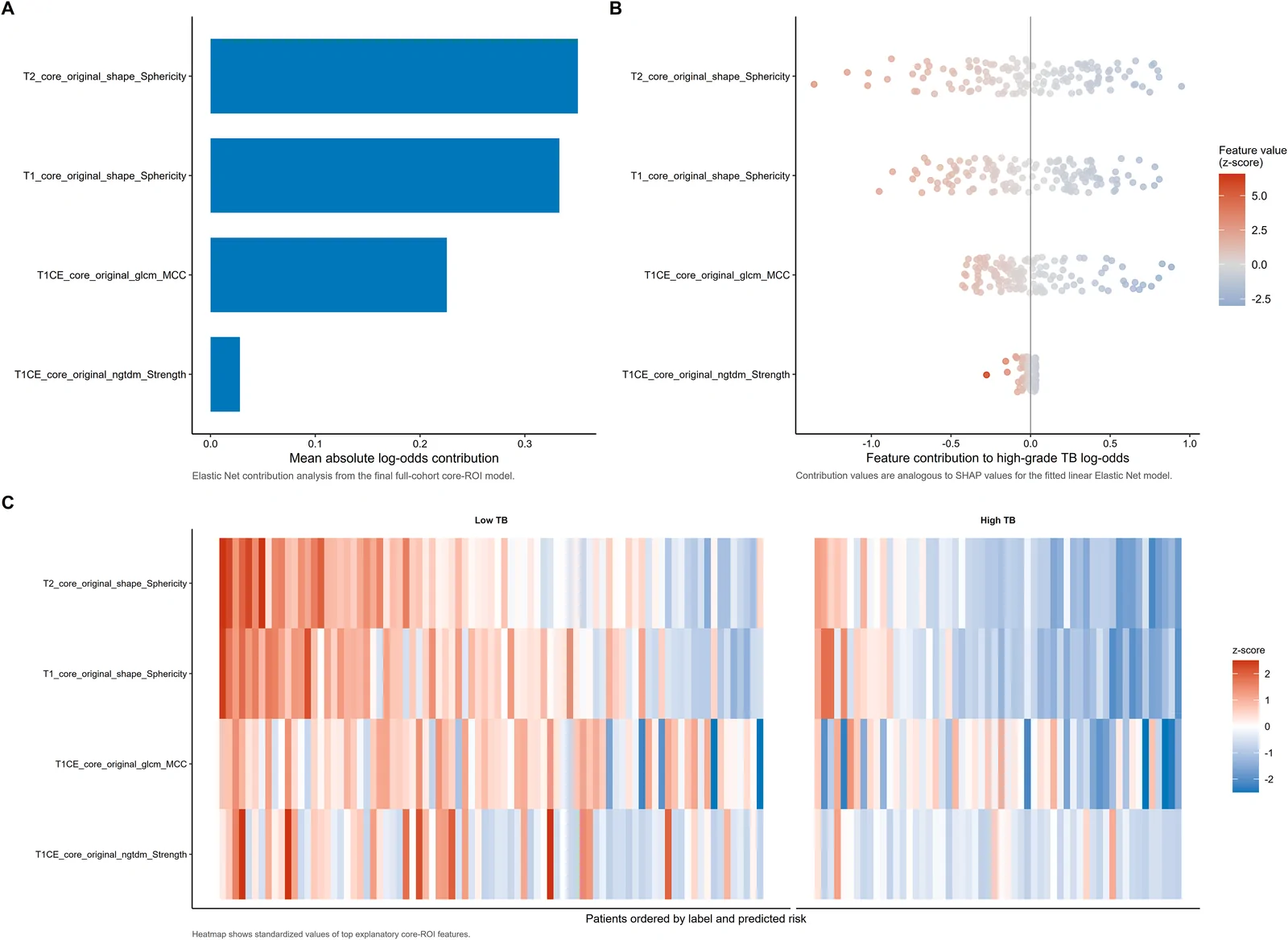
Explanatory features and radiomics phenotype of the primary model. **(A)** Ranked mean absolute contribution of the four leading radiomics variables in the full-cohort refit of the selected core Elastic Net model. **(B)** Coefficient-derived contribution summary for the same variables from the full-cohort refit. **(C)** Heatmap of standardized values for these variables in patients with low-grade versus intermediate/high-grade TB.

**Table 4:** Four leading explanatory features in the full-cohort refit of the selected core Elastic Net model.

Feature	Mean absolute contribution
T2 core shape Sphericity	0.351
T1 core shape Sphericity	0.333
T1CE core GLCM MCC	0.226
T1CE core NGTDM Strength	0.028

Mean absolute contribution is the average absolute standardized-feature contribution to model log-odds. These full-cohort refit features are explanatory variables, not independently validated stable biomarkers. GLCM, gray-level co-occurrence matrix; NGTDM, neighboring gray tone difference matrix.

## Discussion

This study demonstrates that multiparametric MRI radiomics can meaningfully distinguish low-grade from intermediate/high-grade tumor budding in rectal cancer before surgery. The tumor-core feature set combined with Elastic Net achieved the highest repeated cross-validation AUC of 0.825 (empirical 2.5th–97.5th percentile range, 0.809–0.839) and a patient-level OOF AUC of 0.829 (95% CI, 0.755–0.896) after 2,000 paired bootstrap replicates. In conditional internal comparisons, the core model showed higher AUCs than the clinical-only, isolated-ring, and retained clinical-radiomics fusion models. Its difference from the expanded ROI was smaller and did not remain statistically significant after adjustment. These comparisons should not be interpreted as definitive evidence of model superiority because model selection used the same repeated cross-validation framework and the bootstrap analysis did not repeat the full model-development pipeline. Overall, the findings support the tumor core as the most informative spatial representation among those retained for TB grading in this cohort.

The intended clinical role of the model is preoperative TB risk stratification in patients with rectal cancer who have not received neoadjuvant treatment and are proceeding to upfront surgery. It offers an imaging-based estimate of TB grade before the resection specimen is available and may help focus review of tumor morphology and local stage. A higher estimated risk should be interpreted as a prompt for closer clinical review, not as a basis for choosing neoadjuvant therapy or independently changing the surgical plan. The model does not address TB after neoadjuvant treatment, because treatment can alter the histological findings used as the reference standard.

The clear advantage of the tumor core over the isolated 2-mm ring also provides an important biological and methodological message. Recent rectal cancer MRI studies have increasingly extended radiomics beyond the tumor boundary. Habitat heterogeneity and peritumoral radiomics improved risk stratification in locally advanced rectal cancer (19), tumoral and peritumoral radiomics supported early T-stage classification (20), intratumoral-peritumoral radiomics improved preoperative prediction of perineural invasion (21), and tumor microenvironment habitat and peritumoral features helped distinguish T1–2 from T3 disease (22). These reports reinforce the value of the peritumoral compartment, but they do not overturn the main result of the present study. Although TB is counted at the invasive front, the MRI phenotype associated with higher TB grade was captured more effectively by the tumor itself than by the narrow external ring. The core contains the integrated macroscopic consequences of tumor cellularity, necrosis, vascular heterogeneity, stromal remodeling, and irregular growth. By contrast, the ring contains a mixture of mesorectal fat, vessels, normal tissue, partial-volume voxels, and variable stromal components. In addition, a fixed 2-mm distance cannot fully represent the invasive zone across tumors with different sizes and morphologies. The present results therefore support the use of a tumor-centered radiomics representation as the primary imaging substrate for TB grading, while peritumoral analysis should be refined rather than simply added as a larger feature block.

The explanatory features further characterize the imaging phenotype detected by the model. T2WI and T1WI core sphericity were among the leading features in the full-cohort refit of the selected model, indicating that tumor geometry carried substantial information. Lower sphericity represents a less compact and more irregular lesion configuration, which is consistent with the complex growth pattern expected in biologically aggressive tumors (23, 24). Contrast-enhanced T1WI GLCM maximal correlation coefficient and NGTDM strength also contributed to the full-cohort refit, providing complementary information about the spatial organization and local intensity differences of enhancing tumor voxels. Together, the features characterize a model-explanatory phenotype marked by tumor geometry and heterogeneous internal organization. They are explanatory features from the full-cohort refit, not confirmed stable biomarkers, and should not be interpreted as direct measurements of tumor-budding cells or epithelial–mesenchymal transition.

The present findings build on previous MRI radiomics studies of TB in rectal cancer (8–18). Earlier reports established that T2WI, multiparametric MRI, diffusion-related features, and machine-learning models can provide preoperative information on TB status, but many were limited by a narrow comparison of sequences, a single tumor-centered or peritumoral definition, or a restricted set of algorithms. In contrast, the present study compared four MRI sequences, three spatial ROI definitions, seven retained feature sets, and four machine-learning algorithms within one internally validated framework. The results suggest that spatial representation matters more than simply increasing algorithmic complexity. Across the tested learners, the tumor-core representation remained robust, and Elastic Net delivered the highest overall performance. Imputation, scaling, feature ranking, and redundancy filtering were performed within the training portion of each cross-validation split. Repeated stratified cross-validation with patient-level OOF probabilities provided a more rigorous basis for discrimination, calibration, confusion-matrix, and DCA analyses than apparent performance alone. The paired 2,000-iteration bootstrap analysis also showed that the observed advantage of the core model was not limited to a single AUC point estimate. Together, these safeguards make the results internally coherent and support a reproducible radiomics workflow for external validation, consistent with recommendations for transparent reporting of medical AI and prediction models (25, 26).

Several limitations should be acknowledged. First, the study was retrospective and single-center, with a modest sample of 139 patients, including 56 with intermediate/high-grade TB; the relatively small number of positive cases may limit the precision and stability of feature selection and model coefficients. Second, the primary model was selected from multiple feature-set and algorithm combinations according to the highest repeated cross-validation AUC. This data-driven selection may introduce optimism into the reported performance, even though preprocessing and feature selection were performed within the training portion of each fold, and a nested cross-validation analysis was not performed. Third, the bootstrap analysis estimated uncertainty from patient-level OOF predictions but did not repeat the complete feature-selection and model-refitting pipeline in every bootstrap sample. The resulting confidence intervals therefore describe sampling uncertainty conditional on the available OOF predictions and do not provide a full optimism-corrected estimate. Fourth, the fixed 2-mm ring is narrower than the native through-plane resolution of several MRI sequences, and alternative ring widths such as 3 or 5 mm were not evaluated. Partial-volume effects, interpolation, and segmentation uncertainty may therefore have contributed to the lower performance of the peritumoral representation; this result should not be interpreted as evidence that the peritumoral compartment is biologically uninformative. Fifth, the clinical reasons why patients with locally advanced or node-positive disease proceeded to upfront surgery were not systematically recorded, which limits characterization of this selected surgical cohort. In addition, the ICC quantified reproducibility of ROI delineation rather than radiomics feature values; feature-level reproducibility was not separately assessed, so the stability of individual features across readers or delineations remains uncertain. Finally, all MRI examinations were acquired using a single institutional workflow and MRI system, and the model has not yet been tested on data from other scanners, institutions, or acquisition protocols. Calibration and decision-curve analysis were based on internal OOF predictions and require confirmation in an independent cohort.

## Conclusion

Multiparametric MRI radiomics features from the tumor core provided promising internal discrimination of low- versus intermediate/high-grade TB among 139 patients with rectal cancer who underwent upfront surgery without neoadjuvant treatment. The core ROI + Elastic Net model was selected as the primary model because it achieved the highest repeated cross-validation AUC of 0.825, while clinical-plus-core model and the other peritumoral or clinical-radiomics fusion sets did not provide additional benefit in this cohort.

## Data Availability

The raw data supporting the conclusions of this article will be made available by the authors, without undue reservation.
