# Supplementary material for: Multiparametric MRI radiomics for preoperative prediction of tumor budding grade in rectal cancer
Source: Front Oncol. 2026 Oct 14;16:1984365. doi: 10.3389/fonc.2026.1984365 (PMC13713355; doi:10.3389/fonc.2026.1984365)
Supplement: Supplementary file 1 [file Table1.docx]

**Supplementary materials**

Supplementary Table S1. Pairwise patient-level bootstrap comparison of AUCs across the seven retained Elastic Net feature-set models.

Bootstrap unit: patient-level mean out-of-fold (OOF) probabilities from 139 patients; 2,000 paired patient-level replicates. Two-sided P values were calculated with an add-one correction from the bootstrap distribution, rounded to three decimal places, and adjusted using the Benjamini–Hochberg method across all 21 pairwise comparisons. AUC differences are Model 1 minus Model 2.

| Model 1 | Model 2 | AUC 1 | AUC 2 | Observed AUC difference | Bootstrap 95% CI | P value | BH-adjusted P value |
| --- | --- | --- | --- | --- | --- | --- | --- |
| Clinical | Core ROI | 0.580 | 0.829 | -0.249 | -0.362 to -0.147 | 0.001 | 0.004 |
| Core ROI | Clinical + expanded | 0.829 | 0.631 | 0.199 | 0.109 to 0.291 | 0.001 | 0.004 |
| Core ROI | Peritumoral ring | 0.829 | 0.657 | 0.173 | 0.096 to 0.248 | 0.001 | 0.004 |
| Expanded ROI | Clinical + expanded | 0.778 | 0.631 | 0.148 | 0.060 to 0.238 | 0.001 | 0.004 |
| Expanded ROI | Peritumoral ring | 0.778 | 0.657 | 0.122 | 0.055 to 0.194 | 0.001 | 0.004 |
| Clinical | Expanded ROI | 0.580 | 0.778 | -0.199 | -0.310 to -0.089 | 0.002 | 0.005 |
| Core ROI | Clinical + core | 0.829 | 0.671 | 0.158 | 0.069 to 0.255 | 0.002 | 0.005 |
| Core ROI | Clinical + core + ring | 0.829 | 0.691 | 0.138 | 0.051 to 0.226 | 0.002 | 0.005 |
| Clinical | Clinical + core + ring | 0.580 | 0.691 | -0.111 | -0.208 to -0.017 | 0.025 | 0.052 |
| Core ROI | Expanded ROI | 0.829 | 0.778 | 0.051 | 0.009 to 0.098 | 0.023 | 0.052 |
| Expanded ROI | Clinical + core | 0.778 | 0.671 | 0.108 | 0.007 to 0.206 | 0.035 | 0.067 |
| Expanded ROI | Clinical + core + ring | 0.778 | 0.691 | 0.087 | 0.001 to 0.173 | 0.050 | 0.087 |
| Clinical | Clinical + core | 0.580 | 0.671 | -0.091 | -0.193 to 0.009 | 0.077 | 0.115 |
| Clinical + expanded | Clinical + core + ring | 0.631 | 0.691 | -0.060 | -0.123 to 0.006 | 0.077 | 0.115 |
| Clinical | Peritumoral ring | 0.580 | 0.657 | -0.077 | -0.199 to 0.045 | 0.224 | 0.313 |
| Clinical | Clinical + expanded | 0.580 | 0.631 | -0.051 | -0.140 to 0.042 | 0.271 | 0.356 |
| Clinical + core | Clinical + expanded | 0.671 | 0.631 | 0.040 | -0.038 to 0.117 | 0.305 | 0.377 |
| Peritumoral ring | Clinical + core + ring | 0.657 | 0.691 | -0.034 | -0.120 to 0.050 | 0.465 | 0.542 |
| Peritumoral ring | Clinical + expanded | 0.657 | 0.631 | 0.026 | -0.067 to 0.110 | 0.542 | 0.569 |
| Clinical + core | Clinical + core + ring | 0.671 | 0.691 | -0.020 | -0.084 to 0.040 | 0.527 | 0.569 |
| Peritumoral ring | Clinical + core | 0.657 | 0.671 | -0.014 | -0.117 to 0.086 | 0.792 | 0.792 |

Supplementary Table S2. Image preprocessing and radiomics extraction parameters. Multiphase MRI volumes were rigidly registered and resampled to an isotropic voxel spacing of 1 × 1 × 1 mm³ during preprocessing using SimpleITK v2.4.1.

| Parameter | Final analysis setting |
| --- | --- |
| Software | PyRadiomics v3.1.0; SimpleITK v2.4.1 |
| Image registration | Voxel-based rigid registration of multiphase MRI using a prespecified reference volume |
| Resampling | Preprocessing resampling to 1 × 1 × 1 mm³; no additional resampling during feature extraction (resampledPixelSpacing=None) |
| Interpolation | B-spline interpolation for images; nearest-neighbor interpolation for ROI masks |
| Intensity normalization | For nonzero voxels, m = median(x), s = 1.4826 × MAD(x), and x′ = (x − m) / s; standard deviation was used if the MAD-based scale was unavailable |
| Gray-level discretization | Fixed gray-level bin width of 25 |
| Image type | Original image; no wavelet or Laplacian-of-Gaussian filtered images |
| Extraction settings | label=1; correctMask=True; minimumROIDimensions=2 |
| Minimum ROI voxel count | No independent minimum voxel-count threshold |
| Feature families | First order, shape, GLCM, GLRLM, GLSZM, GLDM, and NGTDM |
| Final feature table | 1,300 variables, including clinical and radiomics variables |
| Mask adjudication | When the two radiologists disagreed, a third senior radiologist with 20 years of experience adjudicated the final mask |

Supplementary Table S3. Representative decision-curve analysis values. Net benefit was calculated from patient-level mean OOF probabilities. The illustrative clinical action is intensified multidisciplinary risk review for patients whose predicted probability of intermediate/high-grade TB exceeds the selected threshold; the threshold is not intended to directly determine neoadjuvant therapy or surgical approach.

| Threshold probability | Core Elastic Net | Treat all | Treat none |
| --- | --- | --- | --- |
| 0.10 | 0.3389 | 0.3365 | 0 |
| 0.20 | 0.2734 | 0.2536 | 0 |
| 0.30 | 0.2405 | 0.1470 | 0 |
| 0.40 | 0.2182 | 0.0048 | 0 |
| 0.50 | 0.1655 | −0.1942 | 0 |

Supplementary Table S4. Training-fold candidate-screening frequency of core ROI radiomics features.

The core feature set was screened separately within each of 25 training folds from five repetitions of stratified five-fold cross-validation. Frequencies count how often a feature was retained after outcome-correlation ranking and redundancy filtering (maximum 20 radiomics candidates before redundancy filtering). This is a descriptive internal screening summary, not the frequency of nonzero Elastic Net coefficients, formal stability selection, biological validation, or evidence of external stability. Features are ordered by decreasing frequency.

| **Feature** | **Training folds retained (n/25)** | **Frequency (%)** |
| --- | --- | --- |
| DWI \| core ROI \| shape Sphericity | 25/25 | 100 |
| T1CE \| core ROI \| shape MajorAxisLength | 25/25 | 100 |
| T1CE \| core ROI \| shape Maximum2DDiameterColumn | 25/25 | 100 |
| T1 \| core ROI \| shape Sphericity | 25/25 | 100 |
| T2 \| core ROI \| shape Sphericity | 25/25 | 100 |
| T1 \| core ROI \| shape MinorAxisLength | 24/25 | 96 |
| T1CE \| core ROI \| glcm MCC | 23/25 | 92 |
| T1CE \| core ROI \| shape MinorAxisLength | 23/25 | 92 |
| T1 \| core ROI \| shape Maximum2DDiameterRow | 20/25 | 80 |
| T1CE \| core ROI \| shape Sphericity | 19/25 | 76 |
| T1 \| core ROI \| shape MajorAxisLength | 19/25 | 76 |
| T1CE \| core ROI \| ngtdm Strength | 17/25 | 68 |
| T2 \| core ROI \| shape MajorAxisLength | 17/25 | 68 |
| DWI \| core ROI \| shape MajorAxisLength | 16/25 | 64 |
| T1CE \| core ROI \| ngtdm Coarseness | 15/25 | 60 |
| T1CE \| core ROI \| shape Maximum2DDiameterSlice | 10/25 | 40 |
| DWI \| core ROI \| shape MinorAxisLength | 8/25 | 32 |
| T1CE \| core ROI \| glcm Imc1 | 7/25 | 28 |
| T1CE \| core ROI \| shape LeastAxisLength | 5/25 | 20 |
| T1 \| core ROI \| shape Maximum2DDiameterColumn | 5/25 | 20 |
| DWI \| core ROI \| shape LeastAxisLength | 4/25 | 16 |
| T1CE \| core ROI \| shape SurfaceArea | 4/25 | 16 |
| T1 \| core ROI \| shape Maximum2DDiameterSlice | 4/25 | 16 |
| T2 \| core ROI \| shape Maximum2DDiameterColumn | 4/25 | 16 |
| DWI \| core ROI \| glszm SmallAreaLowGrayLevelEmphasis | 3/25 | 12 |
| DWI \| core ROI \| shape Maximum2DDiameterRow | 3/25 | 12 |
| T2 \| core ROI \| shape Elongation | 3/25 | 12 |
| DWI \| core ROI \| shape Maximum2DDiameterSlice | 2/25 | 8 |
| T1 \| core ROI \| shape Maximum3DDiameter | 2/25 | 8 |
| T1 \| core ROI \| shape SurfaceVolumeRatio | 2/25 | 8 |
| T2 \| core ROI \| glcm SumSquares | 2/25 | 8 |
| DWI \| core ROI \| glrlm LongRunLowGrayLevelEmphasis | 1/25 | 4 |
| DWI \| core ROI \| ngtdm Coarseness | 1/25 | 4 |
| DWI \| core ROI \| shape Maximum3DDiameter | 1/25 | 4 |
| T2 \| core ROI \| glcm DifferenceVariance | 1/25 | 4 |
| T2 \| core ROI \| glrlm LongRunHighGrayLevelEmphasis | 1/25 | 4 |
| T2 \| core ROI \| glszm SmallAreaHighGrayLevelEmphasis | 1/25 | 4 |
| T2 \| core ROI \| ngtdm Complexity | 1/25 | 4 |
| T2 \| core ROI \| shape SurfaceArea | 1/25 | 4 |
| T2 \| core ROI \| shape SurfaceVolumeRatio | 1/25 | 4 |
